# Supplementary material for: Tedizolid-Cyclodextrin System as Delayed-Release Drug Delivery with Antibacterial Activity
Source: Int J Mol Sci. 2020 Dec 24;22(1):115. doi: 10.3390/ijms22010115 (PMC7795824; doi:10.3390/ijms22010115)
Supplement: Supplementary file 1 [file ijms-22-00115-s001.pdf]

Supplementary material

Table S1. Summary of results for validation parameters of the HPLC-DAD method at pH 1.2, pH 4.5 and pH 6.8

Parameter	Dissolution and permeability studies		
	pH 1.2	pH 4.5	pH 6.8
Selectivity			
Peak symmetry factor (0.8-1.5 required)	1.3	1.1	1.1
Absence of interfering substances	confirmed	confirmed	confirmed
Limit of detection, LOD [mg mL^{-1}]	0.0014	0.0003	0.0004
Limit of quantification, LOQ [mg mL^{-1}]	0.0042	0.0011	0.0013
Linearity y = ax + b			
a ± S_a	143.89±2.55	58.76±3.06	60.78±1.39
b ± S_b	insignificant	insignificant	insignificant
Correlation coefficient (*r*)	0.9998	0.9981	0.9997
Range of linearity [mg mL^{-1}]	0.006 – 0.068	0.001 – 0.006	0.002 – 0.021
Accuracy			
Recovery (95-105% requires) [%]	101.4	97.6	97.5
Precision			
Concentration [mg mL^{-1}]	0.0680	0.0060	0.0210
Average of 6 injections [mg mL^{-1}]	0.0689	0.0059	0.0205
SD	0.0017	0.0002	0.0003
RSD (<5% required) [%]	2.47	3.39	1.46

where S_D is the average of standard deviations of determinations in the lower range of linearity and *a* is the directional coefficient of the plotted linear function; *S*a standard deviation of slope; *S*b standard deviation of intercept, *t*, calculated values of Student's t-test, $t_{\alpha,f}$ = 2.228 critical values of Student's test for degrees of freedom *f* = 10 and significance level α = 0.05.
